# Development of a Framework for Generating Driving Safety Assessment Scenarios for Automated Vehicles

**DOI:** 10.3390/s22166031

**Published:** 2022-08-12

**Authors:** Woori Ko, Sangmin Park, Jaewoong Yun, Sungho Park, Ilsoo Yun

**Affiliations:** 1 Department of Transportation Engineering, Ajou University, Suwon 16499, Korea; 2 Department of Road Transport Research, The Korea Transport Institute, Sejong 30147, Korea; 3 Department of Mobility, TÜV SÜD Korea Ltd., Seoul 07326, Korea; 4 Department of Transportation System Engineering, Ajou University, Suwon 16499, Korea

**Keywords:** framework, driving safety, assessment, scenarios, automated vehicles

## Abstract

Despite the technological advances in automated driving systems, traffic accidents involving automated vehicles (AVs) continue to occur, raising concerns over the safety and reliability of automated driving. For the smooth commercialization of AVs, it is necessary to systematically assess the driving safety of AVs under the various situations that they potentially face. In this context, these various situations are mostly implemented by using systematically developed scenarios. In accordance with this need, a scenario generation framework for the assessment of the driving safety of AVs is proposed by this study. The proposed framework provides a unified form of assessment with key components for each scenario stage to facilitate systematization and objectivity. The performance of the driving safety assessment scenarios generated within the proposed framework was verified. Traffic accident report data were used for verification, and the usefulness of the proposed framework was confirmed by generating a set of scenarios, ranging from functional scenarios to test cases. The scenario generation framework proposed in this study can be used to provide sustainable scenarios. In addition, from this, it is possible to create assessment scenarios for all road types and various assessment spaces, such as simulations, proving grounds, and real roads.

## 1. Introduction

Many companies worldwide are working to develop reliable automated vehicles (Avs). As a result, AV technology has grown remarkably. As proof of this, AVs are already driving on real roads in many cities worldwide. The most representative AVs driving on real roads are the Tesla Autopilot and Cadillac Super Cruise. AVs developed by Waymo, Uber, and other companies are also being experimented with, on real roads [1,2,3]. Recently, the Honda Motor Company introduced the “Legend”, with level three automated driving technology for certain conditions, such as congestion situations [4]. Mercedes-Benz was the first to receive internationally valid certification for a level three automated driving technology, launching the “Drive Pilot” system to support this technology [5].

Despite advances in automated driving technology, AV-related traffic accidents continue to occur. AV traffic accident news reports lead to concerns regarding the safety and reliability of automated driving systems (ADSs). In fact, from analyzing the contexts used by newspapers published in Singapore over a period of approximately 67 months, it was found that AV safety, the economy, and the use of personal data were the main issues of debate [6]. Therefore, for the smooth commercialization of AVs, it is necessary to develop a method to ensure AV safety.

To ensure AV safety, it is necessary to secure driving safety among the various AV safety fields. Crash safety and functional safety can be sufficiently secured through the development of automated driving technologies. However, research on driving safety, defined as a vehicle recognizing, judging, and reacting to the driving environment on its own in all situations encountered while driving, remains lacking. When driving safety is secured, an AV can drive harmoniously while complying with traffic laws and minimizing conflicts with the surrounding vehicles.

To assess driving safety, there is a need for a scenario-based assessment that is able to reflect various driving situations. A scenario-based assessment has the advantage of being adjustable with regard to difficulty and can be used in various assessment spaces, including simulations and proving grounds (PGs), as well as real roads [7,8]. In addition, a scenario-based approach can meticulously assess various situations because it can handle all well-designed scenarios [9].

In view of the advantages of the scenario-based approach, this study proposes a scenario generation framework for functional scenarios, logical scenarios, concrete scenarios, and test cases commonly used to assess driving safety. The proposed framework for creating driving safety scenarios is designed to systematically and diversely generate assessment scenarios.

Existing scenario-based research trends and studies are reviewed in Section 2. Section 3 presents an overview of the development of the driving safety scenario generation framework, functional scenario generation framework, logical scenario generation framework, concrete scenario generation framework, and test case generation framework. In Section 4, the performance of the proposed scenario framework is verified by creating a scenario using the proposed framework and implementing the generated scenario using a simulation. Section 5 presents the conclusions and scope for future research.

## 2. Literature Review

### 2.1. Related Research

The research trends related to AV assessment scenarios were reviewed and are discussed below. The PEGASUS project, a representative scenario-based approach, presents scenario-based verification for automated driving functions in three stages: functional, logical, and concrete. In this approach, a functional scenario is written in natural language to describe and explain road networks, static objects, dynamic objects, environmental conditions, and situations [10,11]. A logical scenario is a scenario that provides the parameter types and a range of state values used for the expression of scenario implementation, and a concrete scenario is a scenario in which specific experimental values of the parameters are specified for actual experiments [10,11]. In the PEGASUS project, as one moves from the functional scenario to the concrete scenario, the level of abstraction decreases, and the number of scenarios increases [10,11]. In addition to defining the scenario system, the PEGASUS project developed and presented a six-layer model. The six-layer model defined the structure of the scenario parameters and systematically classified and managed them according to their characteristics. Each layer corresponded to road-level elements, traffic infrastructure, and temporary modifications to the road-level and traffic infrastructure elements, objects, environment, and digital information [12].

Subsequently, the Association for the Standardization of Automation and Measuring Systems (ASAM) announced the OpenX series for managing static and dynamic simulation applications related to automated driving [13]. Among them, OpenCRG, OpenDRIVE, and OpenSCENARIO are particularly closely related to the scenario approach. Each provides standards for the description of the road surface, the static environment around the road, and the dynamic environment of the traffic participants, respectively. Specifically, OpenCRG defines a file format for the description of road surfaces and performs realistic 3D-rendering of road surfaces [14]. OpenDRIVE defines a standard for describing the static objects around road networks so that advanced driver assistant systems (ADASs) and AVs can be developed and verified through simulation [15]. For example, descriptions of objects such as signs, signals, tunnels, and railways are defined in OpenDRIVE. Finally, OpenSCENARIO defines a file format to describe dynamic objects, such as maneuvers and trajectories related to the weather or traffic participants [16,17]. Similar to OpenDRIVE, OpenSCENARIO tests can be used to verify and certify the safety of ADASs and AVs [17].

The “Validation Method for Automated Driving” of the United Nations Economic Commission for Europe (UNECE) Working Party 29 (WP.29) disclosed a scenario developed by “subgroup 1” through the publication of a new assessment/test method (NATM) master document. The scenario assessed highway chauffeur ADSs and contained pictures and general descriptions of 20 scenarios [18]. In addition to the scenario development examples, this document also presented simulation/virtual tests, track tests, real-world tests, audits/assessments, and in-service monitoring as five methods for verifying safety assessments for AVs [18].

### 2.2. Prior Studies

In this study, prior studies on automated driving scenario generation were considered. So et al. [19] generated functional scenarios for the safety assessment of AVs on urban roads. After capturing the words frequently mentioned in the accident situation descriptions from traffic accident data, they weighted them based on the frequency of expression of each word and then categorized the words by type. A scenario was structured by combining the categorized words to construct urban road and intersection sections. The generated functional scenario was expressed in terms of locations, maneuvers, and a simplified two-dimensional explanatory diagram, along with a textual explanation.

Park et al. [20] developed a scenario-mining methodology for applying natural language processing to accident situation descriptions from traffic accident data to generate assessment scenarios for Avs. In this methodology, traffic accident data were classified as major features using the “frequency-inverse document frequency” term. Then, functional scenarios were created for urban roads through a scenario-mining technique that created scenarios according to the categorized features. These scenarios were expressed using two-dimensional explanatory diagrams, maneuvers, provoking events, and textual explanations.

Nalic et al. [21] argued that a simulation-based test in a complex virtual environment is necessary to derive effective results for automated driving, and they performed a co-simulation of PTV Vissim and IPG CarMaker. In this study, a stress testing method (STM) was developed using Vissim to create and augment scenarios that mimic human driving errors by manipulating traffic participants near the vehicle being tested. The functional scenario generated through STM expressed the driving trajectory for each time period using two-dimensional explanatory diagrams.

Gelder et al. [22] proposed an eight-step safety assessment framework for the safety design of AVs. After generating candidate scenarios using real-road driving data, the parts biased toward the tail of the probability distribution of the candidate scenarios were created as the test scenarios. The generated scenarios showed a two-dimensional schematic diagram and the longitudinal and lateral activities of each scenario participant in parallel according to the time period.

Zhu et al. [23] proposed an optimization searching (OS) method to efficiently search for functional scenarios in a large logical space. There were five modules in the OS method for performing parameter movement direction determination, movement probability calculation, movement step determination, repetitive experimental avoidance, and scenario data clustering. By verifying an adaptive cruise control (ACC) algorithm using the proposed OS method, it was confirmed that the risk parameter space could be quickly found in a given logical scenario and that the test efficiency was effectively increased.

Menzel et al. [24] proposed an approach for converting a keyword-based scenario description from a functional scenario into a logical scenario using two steps. First, in a “scenario-detailing” step, the description in the functional scenario was converted into a parameter space representation according to a five-layer model. Subsequently, it was converted to the OpenX format in a “format-conversion” step. According to their approach, logical scenarios written in the OpenX format could be appropriately visualized and verified through an OpenDRIVE-viewer or virtual test drive.

Piazzoni et al. [25] developed a framework for the virtual scenario-based testing of an AV (ViSTA). The ViSTA framework used a mix of manual and automatic generation to design test cases, aiming to address specific challenges that AVs must overcome in a virtual test environment. In the manual generation design, various complete scenarios were created. In the automatic generation design, each test case was transformed into a machine-readable format, and the visualized results were calculated.

Xinxin et al. [26] performed object detection and tracking by analyzing actual traffic accident image data with a deep neural network module to extract critical scenarios. The extracted scenarios were recreated in a simulator and visualized in three dimensions. A method for assessing the safety score was presented for the scenarios generated in this manner, based on CARLA and MATLAB, using the OpenX format.

Nalic et al. [27] summarized 86 papers related to ADS-assessment scenarios. The functional and logical scenarios were divided into expert-knowledge and data-driven types. In addition, it was confirmed that a concrete scenario could be created by combining and sampling the parameter distribution of the logical scenario, according to the data. Finally, the most common and widely used safety metrics for assessing the importance of the generated scenarios were presented.

### 2.3. Summary

The stage and layer model of a scenario, defined in the PEGASUS project, is systematic. However, the PEGASUS project only presented the overall scenario development framework; the detailed methodologies were not disclosed. Therefore, there is a limitation in that it is difficult to create a scenario directly with the scenario development framework within the PEGASUS project. Therefore, in this study, the scenario stage in the widely used PEGASUS project is utilized, but an additional detailed procedure is defined to develop a specific framework for directly creating a scenario. Additionally, we redefined the six-layer model according to the situation in Korea. Our intent is to configure the newly defined layer to include all of the potential scenarios in consideration of the domestic specifics.

The ASAM standard is a format for expressing scenarios, and extensible markup language (XML) codes are usually used in machine-readable structures. Using the ASAM standard has significant advantages with regard to sharing the results from the scenario generation in a unified format. However, a scenario written in the XML code is difficult for human raters to intuitively understand. Therefore, this study proposes a unified format for generating scenarios in an easy-to-understand format for the human raters who will perform the actual assessments, and it attempts to differentiate them by performing the process of converting the scenarios to the format of the ASAM standard.

The NATM master document presents various methodologies for generating scenarios and scenario examples, but only for functional scenarios. This study proposes a framework that generates not only functional scenarios but also logical, concrete, and test scenarios.

Various scenario generation methodologies have been presented in prior studies. Some studies focused on specific scenario stages, such as the functional scenario, but other studies considered the overall scenario generation process. However, the formats of the generated scenarios were all different. Generally, a functional scenario consists of a simple schematic diagram and a textual description for explaining the situation universally. However, schematic diagrams are information-implicit, making it difficult to understand the exact situation. It is very cumbersome and time-consuming to read all of the numerous texts for the contextual descriptions in text form. Therefore, in this study, to unify the scenario writing format, the components that should be included in each scenario stage, from the functional scenario to the test case, were coded. In addition, in prior studies, there were no defined forms for the logical scenarios. Therefore, in this study, a logical scenario writing format was developed and verified.

## 3. Scenario Generation Framework

### 3.1. Overview

In this study, a framework for generating assessment scenarios for AV driving safety was proposed, based on the scenario stages (functional, logical, and concrete) and the six layers of the PEGASUS project. The scenario stages (functional, logical, and concrete) and the six layers of the PEGASUS project have been utilized by various studies for AV scenario creation as a representative scenario-based approach, with systematic scenario creation being possible. In the PEGASUS project, a scenario is presented in only three stages (functional, logical, and concrete), but in this study, a test case is added at the end and is expressed, resulting in a total of four stages.

Therefore, to utilize the basic methodology proposed by the PEGASUS project, this study selected a more basic direction to secure systematicity and objectivity within the scenarios generated through the proposed framework. Here, systematicity denotes that anyone can easily generate a scenario by systematically expressing detailed procedures through a scenario generation framework. In addition, according to the definition of each scenario stage, the system was configured such that the generation of a subsequent scenario was possible after the generation of the preceding scenario was completed. In addition to the overall research system, the scenario-staged system was as follows. Specifically, for the functional scenario generation, a scenario was generated by combining geometries, traffic conditions, driving behaviors, surrounding objects, and weather conditions. For logical scenario generation, the parameters representing the situation were represented by the six PEGASUS layers to enable systematic management.

In this study, the term objectivity refers to including objectively convincing components for the step-by-step scenario-writing form. As a generated scenario should be expressed in a unified form, for an effective approach, it should be possible to do so even when the scenario is generated from data obtained from various sources such as traffic cameras, AV sensors, and traffic accident report data. Therefore, we attempted to secure objectivity by defining the essential components. In addition, the basic direction for securing objectivity was established by synthesizing the results of existing research through a review of previous studies and by supplementing the detailed procedures for framework development.

### 3.2. Functional Scenario Generation Framework and Form

A functional scenario is a scenario with a high degree of abstraction, and it depicts possible situations on a real road in the form of a schematic diagram and brief text [18,19,20,21]. However, as the assessment of driving safety is very complex and varies with time, then if the form of an existing functional scenario is used as-is, it will be difficult to generate a scenario for a driving safety assessment. Therefore, in this study, the functional scenario generation framework and its components are defined by extending the format of the existing functional scenario.

A component refers to a set of information that is necessary to effectively create a functional scenario. The components of the functional scenario were defined as the scenario type, scenario location, scenario purpose, overall situation diagram, scenario description, brief geometry, location of each moving object, movement of each moving object, occurrence situation, data source, scenario writer, and basis for deriving the scenario. In this study, an entire framework was developed by coding the components. The developed functional scenario framework made it easy to search for a specific situation. Moreover, it was possible to formulate functional scenarios from various sources of data, such as traffic camera data, AV sensor data, and traffic accident report data, so as to provide a systematic approach. The functional scenario generation form developed according to the components defined in this study is described in greater detail below.

### 3.3. Logical Scenario Generation Framework and Form

The logical scenario is the stage in which the types and ranges of the various parameters are defined so that the situation can be expressed more clearly than in the functional scenario. In addition, the number of components necessary to define the situation is greater in this case than in the functional scenario. To this end, in this study, and based on the six layers of the PEGASUS project, the detailed components were specifically defined and a system for managing them was developed. This had the advantage of efficiently managing the scenario parameters for each component.

The definition of the PEGASUS project was not used in this study to express a more detailed driving safety situation and create it as a scenario. In this study, Layer 1 consisted of the two-dimensional road shape, surface, and road markings as plane data. Layer 2 comprised three-dimensional infrastructure data, representing road structures, three-dimensional road facilities, and transportation facilities. Layer 3 comprised variable and temporary facilities. Layer 4 represented characteristics such as the speeds and distances of objects potentially affecting driving safety in certain scenarios, such as vehicles, pedestrians, and motorcycles. Layer 5 comprised ambient environment data, such as data concerning illuminance, weather, and visibility. Layer 6 comprised digital data and items affecting AV sensors, vehicle-to-everything communication, and digital maps. Table 1 lists the new six layers as defined in this study.

Based on the six-layer model newly defined in this study, a form was developed for representing logical scenarios. The developed form for the logical scenarios included all three standard definition parameters so that it could be easily converted to ASAM’s OpenCRG, OpenDRIVE, and OpenSCENARIO functions. In general, the logical scenario had a list of parameters requiring assessments and minimum and maximum values as components. In addition, for defining the data types and increase/decrease values, we referred to the selections of the experimental values. As the individual parameters were derived by analyzing various manuals, laws, standards, and data, it was necessary to define the sources of the setting ranges for each parameter together. Here, the components of a logical scenario were the parameter list, data type, minimum value, maximum value, increase/decrease value, and the range-setting source. A form reflecting the relevant components is presented below.

### 3.4. Concrete Scenario Generation Framework

The concrete scenario specifies the experimental values for the ranges of all parameters defined in the logical scenario. Therefore, the component was defined in the concrete scenario by designating an experimental value to the component in the logical scenario. In this study, the format of the concrete scenario was not newly defined; rather, the internationally accepted OpenX standard was used.

The approach selection was performed as the first step in generating a concrete scenario. There are two main types of approaches, testing-based and falsification-based [28]. The testing-based scenario selection was made through the sampling of the logical scenario parameters. The sampling could follow all-pairs, linear, random, or probability distribution-based sampling. For the all-pairs sampling, the experimental value was selected by considering all possible combinations within the set range. The linear sampling and random sampling were used to select the experimental value by sampling evenly along an arbitrary straight line and randomly within the entire range between the minimum and maximum values of the logical scenario, respectively. The probability-distribution-based sampling sampled the experimental values by applying weights according to the probability of occurrence of the parameters. In falsification-based scenario selection, after calculating the risk or complexity of a created concrete scenario, the scenario was selected according to the calculated value [28]. Therefore, falsification-based selection was effective for finding edge cases.

After the approach was selected, experimental values were set for each parameter, and the numbers of possible combinations of experimental values for each parameter were then calculated. Then, the key performance indicator (KPI) and its threshold were set. In general, a KPI determines whether or not a generated scenario is suitable for the intended assessment purposes. Various KPIs can be used, such as time-to-collision (TTC), time-to-react, post-encroachment time, object and event detection and response, and conflict index. Thereafter, the threshold, i.e., the driving safety assessment criterion, was set to an appropriate value by reviewing the related literature and data. At the end of the concrete scenario generation process, simple mathematical modeling was performed. The modeling utilized a simulator that could be exchanged and converted using the OpenX standard. During modeling, the previously set KPI values were calculated for each concrete scenario. This was because there were a very large number of concrete scenarios and assessing all concrete scenarios would lead to large losses, both in terms of time and money; therefore, it was necessary to determine the suitability of the assessment based on the calculated KPI value.

### 3.5. Test Case Generation Framework

Among the concrete scenarios, a scenario determined to be meaningful and feasible, through assessment-suitability analysis, was defined as a test case. Therefore, the test case used the same components and writing form as those used in the concrete scenario. The test case represents a process of selecting a scenario that requires an assessment based on the KPI and threshold set in the concrete scenario generation procedure. Therefore, the first procedure was to analyze the modeling results of the concrete scenarios. Subsequently, a scenario was selected when certain criteria were met according to the set KPI and threshold. Finally, the selected test case was converted into the OpenX standard format to create a scenario for use in an experiment.

## 4. Scenario Generation Using Proposed Frameworks

### 4.1. Overview

**Figure 1:**
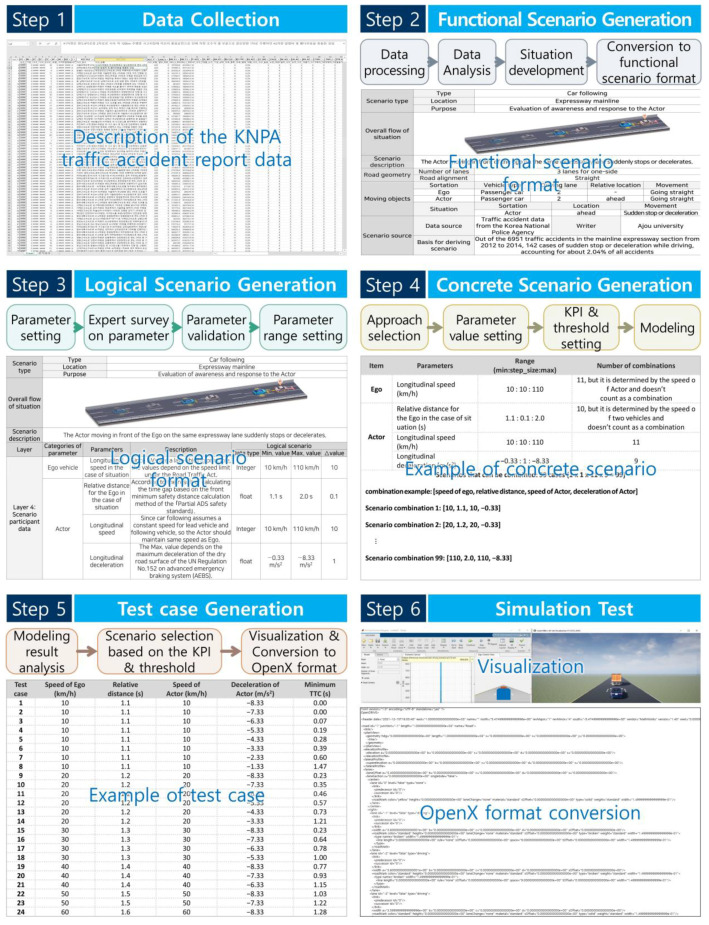
Scenario generation process.

This section describes the generation of the scenarios for the driving safety assessments using the proposed framework, and the verification of the performance of the proposed framework. For the development of a scenario, traffic accident report data from the Korea National Police Agency (KNPA)–one of the various data sources–were used. Based on the proposed scenario framework and data, an entire set of scenarios (from functional scenarios to test cases) was generated. Finally, the usefulness of the framework proposed in this study was confirmed by converting each test case into the OpenX format and implementing it in the simulation model for visualization. The overall scenario creation process using the framework proposed in this study is shown in Figure 1.

### 4.2. Functional Scenario Generation

**Figure 2:**
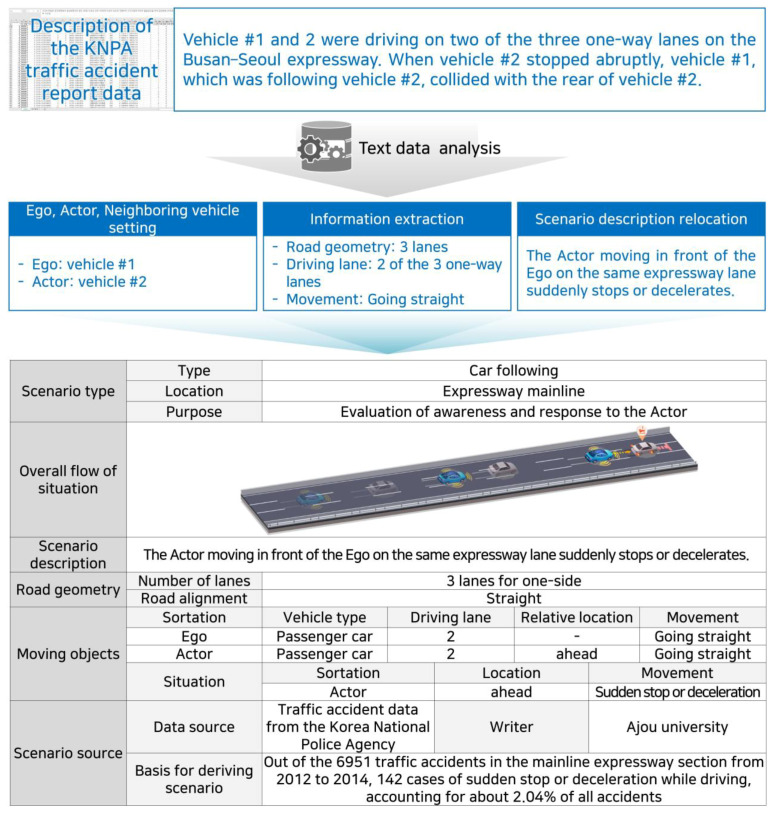
Functional scenario generation result.

The KNPA traffic accident report data included a detailed textual description of each accident. Generally, in a functional scenario, the ego, actors, and neighboring vehicles are first set by analyzing the description text. This is because the circumstances of the occurrence of traffic accidents change according to the ego’s perspective. The purpose of this study was to assess the driving safety of AVs. Therefore, an object (that is, a damaged vehicle) that faces a situation that threatens safe driving, owing to the high probability of an accident while driving in compliance with the law, was set as the ego. Subsequently, the information required for the developed scenario writing form was extracted based on the contents of the description, and the situation that occurred was summarized and rearranged into an easy-to-understand structure to develop a functional scenario. In addition, the frequency of the accident occurrence, severity of the accident, and consideration of existing studies were written as the basis for deriving the scenario. The description example, as expressed according to the developed writing form, is shown in Figure 2. The overall situation can be expressed as “a situation where the ego’s response is required owing to a sudden stop of the preceding actor while following a vehicle.”

### 4.3. Logical Scenario Generation

The logical scenario sets various parameters to more clearly express a situation. In this study, the parameters are set based on the six layers defined herein, by analyzing various literature and data related to the definition of each layer. As a functional scenario, defined in Section 4.2, is a scenario with regards to a motorway (i.e., expressway) main line section, 82 parameters related to the motorway main line section were set, as shown in Table 2. The reason for setting these parameters was to include and display both static and dynamic descriptions of the road surrounding the situation.

For the selected parameters, we used the range defined in the considered literature, or we set the range through the statistical analysis of the data. For example, the Enforcement Rules of the Road Traffic Act state that “the maximum speed is 100 km/h and the minimum speed is 50 km/h on the expressway.” The “Maximum Speed Limit Data by motorway section” is analyzed as follows: “as of August 2021 data, the maximum speed[s] by line [are] 80 km/h, 100 km/h, and 110 km/h.” Therefore, the driving speed range of the ego vehicle can have a maximum of 110 km/h, with increases of 10 km/h from a minimum of 0 km/h. Table 3 lists the minimum, maximum, and increase/decrease values for the 82 parameters according to the developed form.

### 4.4. Concrete Scenario Generation

**Figure 3:**
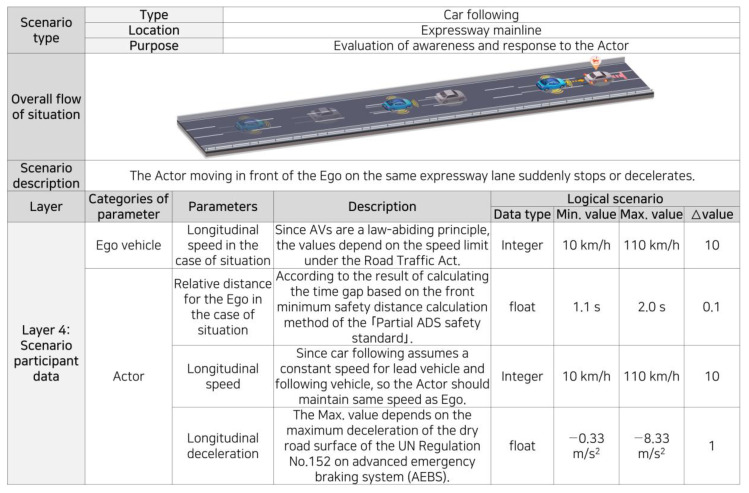
Logical scenario for sudden stops during a car following on the motorway main line.

In principle, the concrete scenario considers all parameters defined within the logical scenario. The parameters must be diverse and numerous to ensure the realism of the scenarios. Various parameters will be considered in the actual test. However, in this section, because the goal is to verify the process of scenario development according to the proposed framework, the case study is relatively simple. We briefly introduce the process of generating a concrete scenario by selecting only four parameters directly affecting the situation, i.e., not all parameters are selected. The parameters concerning a sudden stop while following a vehicle on a main highway section were the speed of the ego, the distance between two vehicles, speed of the actor, and deceleration of the actor. The selected parameters partially reduced the scope of the logical scenario, as shown in Figure 3, according to the situation. Here, because the speed of the ego was based on ACC, it was assumed that the speed was the same as that of the preceding actor. In addition, because the two vehicles had the same speed, it was assumed that the relative distance between them had a constant value according to the speed.

Table 4 shows the results of the concrete scenario creation, showing all possible combinations among the ranges defined in Figure 3, based on the all-pairs method. A total of 99 concrete scenarios were generated.

This study designated KPIs and thresholds for 99 concrete scenarios. The TTC, which refers to the time it takes to crash when two vehicles drive side-by-side in a longitudinal situation, was selected as a KPI. The threshold value for the TTC was set to be less than 1.5 s, based on a literature review [29,30,31,32].

**Figure 4:**
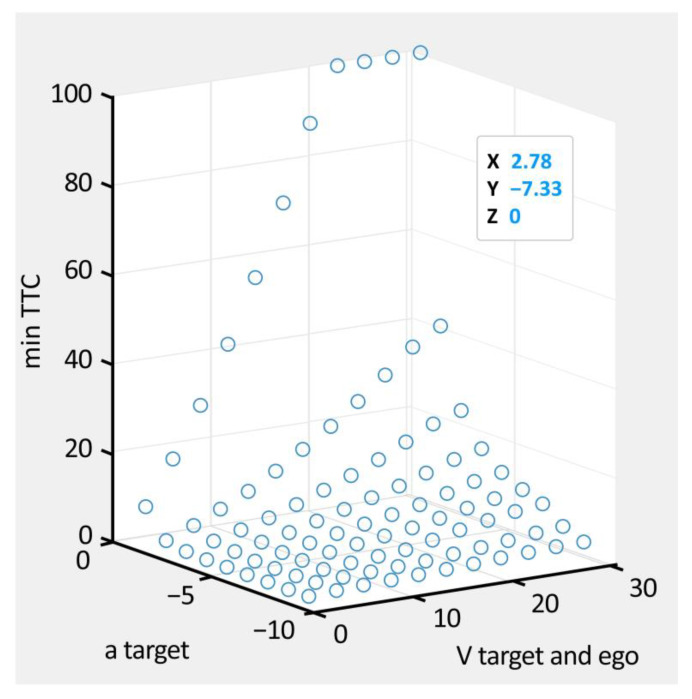
Concrete scenario modeling results.

Subsequently, modeling was performed using MATLAB MathWorks (Natick, MA, USA), a mathematical computing software package. After plotting all the concrete scenarios, only the scenarios satisfying the condition of a TTC of less than 1.5 s were extracted. Figure 4 shows the results from plotting the concrete scenarios of all combinations in the vehicle-following situations based on the TTC.

### 4.5. Test Case Generation

From Table 4, the 99 concrete scenarios were combined; however, only the scenarios satisfying the set KPI and threshold were used as test cases. A test case is a scenario in which the assessment is meaningful owing to the high risk of accidents, and it is extracted based on a plotting graph showing the change in KPI according to the temporal development of the concrete scenario.

**Figure 5:**
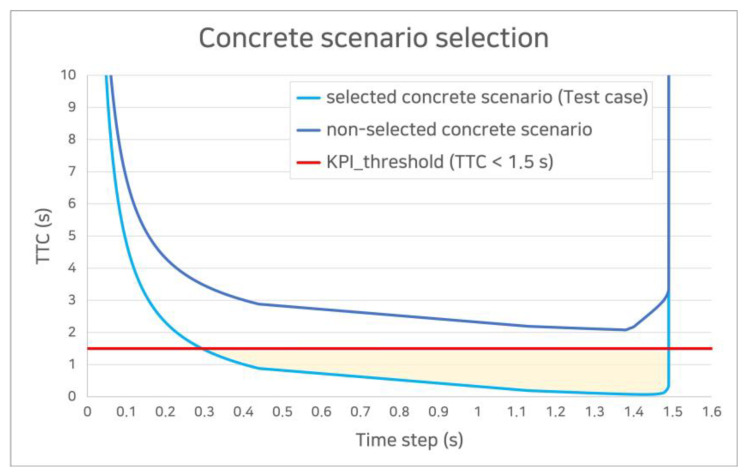
Examples of test case selection.

Figure 5 shows the criteria for selecting a test case from the concrete scenarios. In Figure 5, the sky-blue color line is a case in which a moment that meets the requirement for a TTC of less than 1.5 s exists within the scenario; thus, it is selected as a test case. The navy-blue color line corresponds to a situation in which a test case is not selected because the condition is not satisfied, even for a single moment.

Table 5 lists the results of the combinations of the initial parameter values for the test cases requiring assessment. In this study, 24 test cases were selected. However, as can be seen in Table 5, Test cases 1 and 2 have a minimum TTC of 0.00 s, whereby a collision inevitably occurs. Therefore, 22 test cases, excluding these two test cases, were selected as the initial experimental value combinations for the AV driving safety assessment.

**Figure 6:**
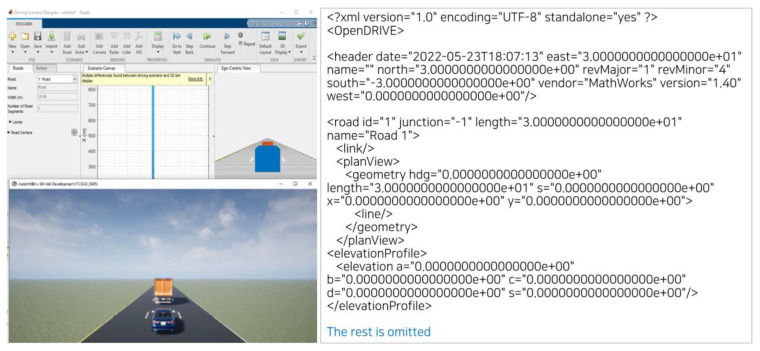
Test case generation result.

Visualization or conversion to the OpenX format was performed for the selected test cases, as shown in Table 5. As a result, it can be confirmed that scenario definition in the OpenX format is possible according to the framework proposed in this study and the developed scenario generation form. In addition, through the visualization of the defined OpenX format using ‘Automated driving toolbox’ in MATLAB, it can be confirmed that the intended scenario was “well-output” within the simulator. Finally, as shown in Figure 6, it was possible to verify the framework and scenario writing style presented in this study. 

## 5. Conclusions

In this study, a scenario-based approach was used to assess the driving safety of AVs. A scenario-based approach is an effective approach that can be used for simulations, PGs, and real roads and is also able to meticulously assess various driving situations by creating various scenarios. Scenarios allow for the systematic assessment of specific driving situations that require an AV to respond without the need to account for all driving behaviors. In addition, this improves overall driving safety as the vehicle manufacturers improve AV safety to pass assessments on AV response. For the assessment scenario, a framework with detailed procedures was defined, with the basic objectives of obtaining systematicity and objectivity. First, in the functional scenario, based on data from various sources, we defined a situation that posed a threat while driving. In the logical scenario, based on laws and data, we set parameters and ranges for describing the situation in more detail. In the concrete scenario, scenarios were diversified through a combination of experimental values within the range set by the logical scenario, and only the scenarios meaningful for assessment, according to the KPI, were extracted as test cases.

This study followed the basic methodology for scenario generation, as defined within the PEGASUS project. However, a detailed scenario generation framework, along with the components and a unified form, were also defined in this study. In addition, six layers were newly defined to develop a scenario writing form. In this study, the performance of the proposed framework was verified by generating a scenario based on the framework. The generated scenario was implemented in the form of OpenX, and the AV driving safety assessment-scenario framework proposed in this study was determined to be suitable.

The proposed framework is not limited to certain roads and can be applied to all roads, so it is expected that it can be utilized to create sustainable scenarios. The various scenarios generated according to the framework proposed in this study can be utilized for AV driving safety assessments. Vehicle manufacturers can improve vehicle safety through the generated assessment scenarios, and assessors can utilize these scenarios within various assessment spaces.

However, the results of this study have a few limitations. For example, the assessment methodology was developed during a time in which AVs have not yet been fully commercialized. Therefore, it is necessary to continuously check and supplement the methodology while AVs are being further commercialized. In addition, in the scenarios generated according to the proposed assessment framework, only the example of a vehicle-following situation on a motorway main line section was presented, and various scenarios need to be generated for all road sections in the future. In this study, the scenarios were verified using simulation software; however, most software has limitations with regard to parameters, so there is a limitation in that the assessment was performed only with a small number of variables. In the future, it is expected that a digital twin will be able to overcome these limitations by providing the same test conditions as those on real roads, allowing for detailed assessment without restrictions and discovering situations that cannot be passed. Lastly, there is a limitation of not being able to verify the assessment framework proposed in this study with actual vehicle assessments. In the case of actual vehicle assessments, it is possible to check which parts of the scenario need to be supplemented, so there is a lack of confidence in the framework proposed in this study owing to the absence of actual vehicle verification. Therefore, we intend to conduct further research in the future to assess AVs and verify the reliability of the proposed assessment methodology.

## Figures and Tables

**Table 1:** New definition of the six-layer approach for this research.

Layer	Keyword	Definition
1	Plane data	All explainable plane data, such as permanent road shape, road surface, and road markings related to road geometry
2	3D infrastructure data	All three-dimensional data, such as permanent road structures and road facilities related to transportation infrastructure
3	Variable facility and temporary facility data	Variable facility data that vary depending on the situation or time zone and temporary facility data, such as temporary road works
4	Scenario participant data	Data related to scenario participants such as object type, number of objects, and object characteristics (speed, distance).
5	Surrounding environment data	Surrounding environment data for indicating driving environment conditions, such as illuminance, weather, and visibility.
6	Digital data	Digital data, such as items and performance data related to automated vehicle (AV) sensors, vehicle-to-everything (V2X) communication, and digital maps

**Table 2:** Logical scenario parameters.

Layers	Categories of Parameter	Parameters					
1	Road Section	Type of Road Section					
	Road alignment	Type of road alignment		Minimum radius of curvature		Minimum length of curvature	
	Road slope	Maximum longitudinal slope					
	Lane	Number of lanes		Minimum lane width	Minimum width of the right shoulder	Minimum width of the left shoulder	
	Road surface marking	Lane color		Lane type		Type of road surface marking except lane	
		Performance level of road surface marking		Color of the lane		Type of color lane	
	Others	Road pothole presence			Maximum speed limit		
2	Road structure	Type of road structure		Type of median strip		Emergency refuge area Presence	
	Road facility	Traffic signal type		Traffic signal installation type		Regulatory sign presence	
		Supplementary sign presence		Type of road lighting facility		Type of bus stop	
3	Variable facility	Type of variable facility		Bus-only lane operation availability	Hard shoulder lane operation availability	Variable speed limit operation availability	
	Temporary facility	Construction and work presence		Construction and work lane		Type of construction and work-related material	
		Accident presence		Accident lane		Accident-related material	
	Others	Temporary parking presence					
4	Ego vehicle	Vehicle type		Initial driving lane		Initial movement	
		Driving lane in the event of a situation		Movement in the event of a situation		Longitudinal speed	
	Actor	Number of Actors		Vehicle type in the case of Actor vehicle		Whether automated driving (AD) is possible in the case of Actor vehicle or not	
		Whether vehicle-to-vehicle (V2V) communication is possible in the case of Actor vehicle		Object type in the case of Actor object		Actor color and material	
		Initial driving/position lane		Initial relative location with respect to that of the Ego vehicle		Initial relative distance from the Ego vehicle	
		Initial movement		Driving/position lane in the event of a situation		Relative location with respect to that of the Ego vehicle in the event of a situation	
		Relative distance from the Ego vehicle in the event of a situation		Movement in the event of a situation		Longitudinal speed	
		Longitudinal acceleration			Lateral lane departure speed		
	Neighboring	Number of Neighboring		Type of Neighboring		Whether AD is possible in the case of neighboring vehicle	
		Whether V2V communication is possible in the case of neighboring vehicle or not		Driving/position lane		Relative location with respect to that of the Ego vehicle	
		Relative distance from the Ego vehicle		Movement		Longitudinal speed	
5	Operational constraints	Density					
	Lighting	Day and night					
	Weather	Weather type		Maximum wind speed		Road condition	
	Visibility	Type of visibility-reducing substance			Density of visibility-reducing substance		
6	AV sensor	Type of sensor			Sensor performance		
	Vehicle-to-everything (V2X) communication	Type of communication			Communication performance		
	Digital map	Type of digital map			Digital map performance		

**Table 3:** Logical scenario generation result.

Layers	Categories of Parameter	Parameters	Description	Logical Scenario			
				Data Type	Min. Value	Max. Value	∆Value
Layer 1: Plane data	Road section	Road section type (Expressway mainline)	-	Categorical	[Road, Shoulder, Flank, Deceleration lane, Acceleration lane]		
	Road alignment	Road alignment type	-	Categorical	[Straight, Curve, Hair pin curve, Transition curve or section]		
		Minimum plane curve radius	Automatically determined according to the road design speed and longitudinal slope.	Integer	-	-	-
		Minimum plane curve length	Automatically determined according to the road intersection angle and road design speed.	Integer	-	-	-
	Road slope	Maximum longitudinal slope	-	float	−6%	6%	1%
	Lanes	Number of lanes	-	Integer	1	6	1
		Minimum lane width	-	float	3.50 m	3.60 m	0.1
		Minimum right shoulder width	Rural area	float	3.50 m	3.60 m	0.1
			Urban area		3.50 m	3.50 m	-
		Minimum left shoulder width	-	float	1.00 m	1.00 m	-
	Road marking	Lane color	-	Categorical	[White, Yellow, Blue]		
		Lane type	-	Categorical	[Solid, Dotted, Double solid, Solid+dotted, Zig-zag solid, Others]		
		Types of road marking other than lanes	-	Categorical	[Not applicable, Arrow, Symbol, Text, Obstacle control, Color lane, Others]		
		Road marking performance level	Road marking painting night reflection performance standard is considered to be 100%, and if it exceeds the standard, input to 100%. (Installation minimum reflection standard—White 240 mcd/(m^2^·Lux), Yellow 150 mcd/(m^2^·Lux), Blue 80 mcd/(m^2^·Lux))	Categorical	0%	100%	10
		Color of color lane	-	Categorical	[Not applicable, Pink, Light green, Green, Blue, Orange]		
		Type of color lane	-	Categorical	[Not applicable, solid, dotted]		
	Others	Whether pothole	-	Categorical	[Not applicable, Presence]		
		Road speed limit	-	Integer	80 km/h	110 km/h	10
Layer 2: 3D data	Road structure	Type of road structure	-	Categorical	[General road, Bridge, Tunnel, Underground road, Overpass, Soundproof facility, Protective facility, Ecological passage, Green area, Emergency parking strip, Rest area, Others]		
		Type of median strip	-	Categorical	[Green area, Protective fence, Concrete barrier, Concrete curb, Others, None]		
		Presence of emergency parking strip	-	Categorical	[Standard, Expandable, None]		
	Road facility	Type of traffic light	-	Categorical	[Vehicle light, Pedestrian light, Bicycle light, Bus lane light, Variable light, None]		
		Traffic light installation type	-	Categorical	[Suspension, longitudinal side pole, lateral side pole, central pole, Signal bridge]		
		Presence of regulator sign	-	Categorical	[Presence, None]		
		Presence of supplementary sign	-	Categorical	[Presence, None]		
		Type of road lighting facility	-	Categorical	[Continuous lighting, Local lighting, No lighting, Tunnel lighting, Others]		
		Type of bus stop	-	Categorical	[Not applicable, Direct bus station, Parallel bus station, Bus stop, Simplified bus stop]		
Layer 3: Variable facility and temporary facility data	Variable facility	Type of variable facility	-	Categorical	[Not applicable, Bus lane, Hard shoulder running, Variable speed limit, Others]		
		Whether bus lane operation	-	Categorical	[Not applicable, Operation, Not operation]		
		Whether hard shoulder running operation	-	Categorical	[Not applicable, Operation, Not operation]		
		Whether variable speed limit operation	-	Categorical	[Not applicable, Operation, Not operation]		
	Temporary facility	Presence of construction and work	-	Categorical	[Not applicable, Presence]		
		Lane of construction and work	-	Integer	1	6	1
		Type of construction and work related materials	-	Categorical	[Not applicable, Work information sign, Arrow sign, Chevron alignment sign, Robot signal, Traffic cone, Safety drum, Temporary lane, Polycarbonate (PC) shield fence, Polyethylene (PE) fence, Steel rail, Others]		
		Presence of accident	-	Categorical	[Not applicable, Presence]		
		Accident lane	-	Integer	1	6	1
		Type of accident related materials	-	Categorical	[Not applicable, Accident vehicle, Debris, Emergency warning triangle, Others]		
	Others	Whether temporary parking	-	Categorical	[Not applicable, Presence]		
Layer 4: Scenario participant data	Ego vehicle	Vehicle type	-	Categorical	[Passenger car, Van, Bus, Truck, Emergency car, Micro electric car, Motorcycle, Others]		
		Initial driving lane	-	Integer	1	6	1
		Initial movement	Lateral movement	Categorical	[Going straight, Cut-in, Cut-out, Cut-through, Others]		
			Longitudinal movement	Categorical	[Constant speed, Accelerating, Decelerating, Stopping, Others]		
		Driving lane in the case of situation	-	Integer	1	6	1
		Movement in the case of situation	Lateral movement	Categorical	[Going straight, Cut-in, Cut-out, Cut-through, Others]		
			Longitudinal movement	Categorical	[Constant speed, Accelerating, Decelerating, Stopping, Others]		
		Longitudinal speed in the case of situation	As Avs are based on law-abiding principles, the values depend on the speed limit under the Road Traffic Act.	Integer	10 km/h	110 km/h	10
	Actor	Number of Actors	-	Integer	0	5	1
		Vehicle type	-	Categorical	[Not applicable, Passenger car, Van, Bus, Truck, Emergency car, Micro electric car, Motorcycle, Others]		
		Whether Actor is AV	-	Categorical	[Level 0, Level 1, Level 2, Level 3, Level 4, Level 5]		
		Whether Actor have V2V communication	Check whether it is possible to provide forward situation information through V2V communication.	Categorical	[Possible, Impossible]		
		Object type	-	Categorical	[Not applicable, Bicycle, Stroller, Personal mobility (PM), Pedestrian, Animal, Falling object, Road object, Others]		
		Color of object in case of falling objects or road objects	Elements for whether obstacle awareness	Categorical	[Not applicable, Contrast to the road surface, Similar to the road surface, Others]		
		Initial driving or located lane	-	Integer	1	6	1
		Initial relative location for the Ego	-	Categorical	[ahead, ahead-left, ahead-right, side-left, side-right, behind, behind-left, behind-right, oncoming, oncoming-left, oncoming-right]		
		Initial longitudinal relative distance for the Ego	According to the [United Nations (UN) Regulation] No.157 5.2.3.3 and the result of previous research and development (R&D) studies.	Float	1.0 s	2.0 s	0.1
		Initial movement	Lateral movement	Categorical	[Going straight, Cut-in, Cut-out, Cut-through, Crossing, Others]		
			Longitudinal movement	Categorical	[Constant speed, Accelerating, Decelerating, Stopping, Walking, Standing, Falling, Bouncing up, Others]		
		Driving or located lane in the case of situation	-	Integer	1	6	1
		Relative location of the Ego in the case of situation	-	Categorical	[ahead, ahead-left, ahead-right, side-left, side-right, behind, behind-left, behind-right, oncoming, oncoming-left, oncoming-right]		
		Relative distance from the Ego in the case of situation	According to the [UN Regulation] No.157 5.2.3.3 and the result of previous R&D studies.	Float	1.0 s	2.0 s	0.1
		Movement in the case of situation	Lateral movement	Categorical	[Going straight, Cut-in, Cut-out, Cut-through, Crossing, Others]		
			Longitudinal movement	Categorical	[Constant speed, Accelerating, Decelerating, Stopping, Walking, Standing, Falling, Bouncing up, Others]		
		Longitudinal speed	The Max. value is based on the 99 percentile of traffic speed data by vehicle detection system (VDS) in 2021.	Integer	0 km/h	130 km/h	10
		Longitudinal acceleration	The Min. value is the maximum possible deceleration result during actual experiment at the Korea Automobile Testing and Research Institute (KARTI), the Max. value is the acceleration calculation value assuming that the arrival time from 0 to 110 km/h in 2 s.	float	−11 m/s^2^	17 m/s^2^	0.1
		Lateral speed when leaving the lane	Calculation value when the lane change time is set to approximately 0.5–5 s with a lane width of 3.5 m	float	0.7 m/s	7 m/s	1
	Neighboring	Number of neighboring vehicles	Neighboring is limited to the case where it is located in eight directions including front, rear, left, right, and diagonal of the Ego.	Integer	0	8	1
		Vehicle or object type	-	Categorical	[Not applicable, Passenger car, Van, Bus, Truck, Emergency car, Micro electric car, Motorcycle, Bicycle, Stroller, Personal mobility (PM), Pedestrian, Animal, Falling object, Road object, Others]		
		Whether Neighboring vehicle is AV	-	Categorical	[Level 0, Level 1, Level 2, Level 3, Level 4, Level 5]		
		Whether Neighboring vehicle has V2V communication	Check whether it is possible to obtain forward situation information through V2V communication.	Categorical	[Possible, Impossible]		
		Driving or located lane	-	Integer	1	6	1
		Relative location of the Ego	-	Categorical	[ahead, ahead-left, ahead-right, side-left, side-right, behind, behind-left, behind-right, oncoming, oncoming-left, oncoming-right]		
		Longitudinal relative distance from the Ego	According to the [UN Regulation] No.157 5.2.3.3 and the result of previous R&D studies.	Float	1.0 s	2.0 s	0.1
		Movement	Lateral movement	Categorical	[Going straight, Cut-in, Cut-out, Cut-through, Crossing, Others]		
			Longitudinal movement	Categorical	[Constant speed, Accelerating, Decelerating, Stopping, Walking, Standing, Falling, Bouncing up, Others]		
		Longitudinal speed	The Max. value is based on the 99 percentile of traffic speed data by VDS in 2021.	Integer	0 km/h	130 km/h	10
Layer 5: Surrounding environment data	Operational constraint	Density	The Max. value is calculated considering the jam density.	Integer	0 passenger car per km per lane (pcpkmpl)	271 pcpkmpl	10
	Lighting	Day and night	-	Categorical	[Day, Night, Sunset, Sunrise]		
	Weather	Weather type	The minimum daily precipitation is 0.1 mm, the average of total precipitation in 2011–2020 based on the https://www.index.go.kr/main.do (accessed on 19 May 2022) is 1622.6 mm, and the maximum daily precipitation in 2011–2020 based on the https://data.kma.go.kr/cmmn/main.do (accessed on 19 May 2022) is 01.9 mm (2 October 2019).	Categorical	[Clear, Cloudy, Snow, Sleet, Rain, Fog, Thunder and lightning, Others]		
		Maximum wind speed	-	float	0 m/s	49 m/s	1
		Road surface condition	-	Categorical	[Dry, Wet, Frozen, Snow on the road, Others]		
	Visibility	Type of visibility reducing substances	-	Categorical	[Not applicable, Fog, Smoke, Smog, Dust and yellow dust]		
		Concentration of visibility reducing substances	In accordance with the comprehensive air-quality index (CAI) standard. The 0–50 section is good, the 51–100 section is normal, the 101–250 section is bad, and the 251–500 section is very bad	Integer	0	500	1
Layer 6: Digital data	Sensor of AV	Type of sensor	-	Categorical	[Camera, Lidar, Radar, Ultrasound, Infrared, Inertial measurement unit (IMU), global positioning system (GPS), Others]		
		Sensor performance related matters	-	Categorical	[Nothing special, Sensor failure, Unrecognition, Positioning error, False positives, Others]		
	V2X communication	Type of communication	-	Categorical	[Vehicle-to-infrastructure (V2I), V2V, Vehicle-to-pedestrian (V2P), Vehicle-to-network (V2N), Vehicle-to-cloud (V2C), Others]		
		Communication performance related matters	-	Categorical	[Nothing special, Latency, Communication error, Communication security risk and hacking, Communication interference, Others]		
	Digital map	Type of digital map	-	Categorical	[High-definition map, Local dynamic map, Others]		
		Digital map related matters	-	Categorical	[Nothing special, Positioning error, Unloadable (Faulty), Unrecognizable, Delayed update, Others]		

**Table 4:** Concrete scenario generation results.

Categories of Parameter	Parameters	Range (min:step_size:max)	Number of Combinations
Ego	Longitudinal speed (km/h)	10:10:110	11, but it is determined by the speed of Actor and does not count as a combination
Actor	Relative distance from the Ego in the case of situation (s)	1.1:0.1:2.0	10, but it is determined by the speed of two vehicles and does not count as a combination
	Longitudinal speed (km/h)	10:10:110	11
	Longitudinal deceleration (m/s^2^)	−0.33:1:−8.33	9
Scenarios that can be combined: 99 cases (1 × 1 × 11 × 9 = 99) <combination example> [speed of Ego, relative distance, speed of Actor, deceleration of Actor] Scenario combination 1: [10, 1.1, 10, −0.33] Scenario combination 2: [20, 1.2, 20, −0.33] ⫶ Scenario combination 10: [100, 2.0, 100, −0.33] Scenario combination 11: [110, 2.0, 110, −0.33] Scenario combination 12: [10, 1.1, 10, −1.33] Scenario combination 13: [20, 1.2, 20, −1.33] ⫶ Scenario combination 99: [110, 2.0, 110, −8.33]			

**Table 5:** Test case generation result.

Test Case	Speed of Ego(km/h)	Relative Distance(s)	Speed of Actor (km/h)	Deceleration of Actor (m/s^2^)	Minimum TTC(s)
1	10	1.1	10	−8.33	0.00
2	10	1.1	10	−7.33	0.00
3	10	1.1	10	−6.33	0.07
4	10	1.1	10	−5.33	0.19
5	10	1.1	10	−4.33	0.28
6	10	1.1	10	−3.33	0.39
7	10	1.1	10	−2.33	0.60
8	10	1.1	10	−1.33	1.47
9	20	1.2	20	−8.33	0.23
10	20	1.2	20	−7.33	0.35
11	20	1.2	20	−6.33	0.46
12	20	1.2	20	−5.33	0.57
13	20	1.2	20	−4.33	0.73
14	20	1.2	20	−3.33	1.21
15	30	1.3	30	−8.33	0.23
16	30	1.3	30	−7.33	0.64
17	30	1.3	30	−6.33	0.78
18	30	1.3	30	−5.33	1.00
19	40	1.4	40	−8.33	0.77
20	40	1.4	40	−7.33	0.93
21	40	1.4	40	−6.33	1.15
22	50	1.5	50	−8.33	1.03
23	50	1.5	50	−7.33	1.22
24	60	1.6	60	−8.33	1.28

## Data Availability

The data used in this study can be made available.
